# Blunt Chest Trauma-Induced Left Internal Mammary Artery Injury Requiring Interventional Radiology Embolization: A Case Report

**DOI:** 10.7759/cureus.109452

**Published:** 2026-05-22

**Authors:** Henry Knox, Rachel Hemsath, Saptarshi Biswas

**Affiliations:** 1 Medicine, Edward Via College of Osteopathic Medicine (VCOM) - Carolinas, Spartanburg, USA; 2 General Surgery, Medical College of Georgia, Augusta, USA; 3 Surgery, Grand Strand Medical Center, Myrtle Beach, USA

**Keywords:** bleeding, blunt chest trauma, internal mammary artery, interventional radiology guided embolization, treatment

## Abstract

Internal mammary artery (IMA) injuries caused by blunt chest trauma are rare and can prove to be fatal; diagnosis is often missed due to the variety of patient presentations. IMA injury can also occur after lower-energy mechanisms, and management standards and treatment guidelines for these injuries remain limited. If left undetected, severe consequences, including life-threatening intrathoracic hemorrhage, may occur. Therefore, prompt and accurate diagnosis is essential following blunt chest trauma. We present a unique case of a 60-year-old male patient who sustained an injury to the left IMA after falling from his bicycle. The patient's hemoglobin and hematocrit were 10.5 g/dL and 29.8%, respectively. His systolic blood pressure decreased from an initial 171 mmHg to the 80s while in the emergency department. Computed tomography (CT) of the chest, abdomen, and pelvis with contrast showed a large subcutaneous hematoma overlying the left pectoralis muscle with a small focus of active extravasation. Urgent coil embolization was done by interventional radiology (IR) to occlude an area of active bleeding arising from a branch of the left internal mammary artery. A post-procedural angiogram revealed resolution of active bleeding. The patient remained neurologically and hemodynamically stable, his hemoglobin stabilized, and he was cleared for discharge home after physical therapy and occupational therapy evaluation. At trauma clinic follow-up, his left chest wall swelling had significantly improved without post-procedural complications. Patients with IMA injuries may initially present with mild symptoms and limited external findings following minor trauma. However, an undiagnosed IMA injury can cause fatal hemorrhage. Clinicians should therefore include IMA injury in the differential diagnosis for patients with blunt anterior chest trauma, enlarging chest wall hematoma, hypotension, or active extravasation on imaging. CT angiography can effectively identify active extravasation and guide definitive angiographic management. As demonstrated in this case, embolization can provide rapid and effective hemorrhage control in selected patients and may avoid the morbidity of open surgical repair.

## Introduction

The internal mammary artery (IMA), also known as the internal thoracic artery, courses along the sternum and is susceptible to injury from blunt chest trauma, although this is rare. IMA injury can cause rapid accumulation of blood in the thoracic cavity because of its proximity to the pleura and mediastinum [1].

Diagnosis is often missed because of variable presentations, especially in patients who are initially hemodynamically stable. Additionally, literature outlining management standards and treatment guidelines for these injuries remains limited. In a review of 5,305 female patients with blunt-force trauma, 94% of injuries were caused by motor vehicle accidents, and only seven were diagnosed with significant breast hematomas requiring invasive intervention, demonstrating the rarity of this presentation [2].

Although rare, an undetected IMA injury can result in life-threatening hemorrhage; therefore, prompt and accurate diagnosis is essential following blunt chest trauma. We present a unique case of a male patient who sustained a left IMA injury after falling from his bicycle and required urgent Interventional Radiology-led embolization.

This case report was previously presented as a poster at the American College of Chest Physicians Annual Meeting in Boston, Massachusetts, on October 9, 2024 [3].

## Case presentation

A 60-year-old male presented to the emergency department (ED) one hour after a bicycle accident. The patient was riding his bike when his foot got caught in the bike, and he was thrown over the handlebars. He was wearing a helmet during the accident. Although the patient admitted to having struck his head during this event, he denied any loss of consciousness, headache, numbness, tingling, weakness, injury to his abdomen, neck pain, back pain, nausea, vomiting, diarrhea, or blood in his urine. He also denied any dizziness or lightheadedness prior to the fall. Prior to ED arrival, he was seen at an Urgent Care Center with an enlarging hematoma to the left chest and a left wrist injury. He denied any other modifying factors or associated signs or symptoms. 

The patient was initially evaluated by ED physicians and was subsequently upgraded to a trauma consult. Primary and secondary surveys were completed according to Advanced Trauma Life Support (ATLS) protocol. Initial vital signs included blood pressure of 171/93 mmHg, heart rate of 76 beats per minute, temperature of 97.6 ^o^F, respiratory rate of 16 breaths per minute, and oxygen saturation of 98%. Laboratory values included a white blood cell count of 12.1 × 10³/µL, red blood cell count of 4.22 × 10⁶/µL, hematocrit of 41.0%, glucose of 122 mg/dL, blood urea nitrogen of 23 mg/dL, potassium of 5.4 mmol/L, prothrombin time of 14.8 seconds, and an international normalized ratio of 1.2.

Past medical history included hypertension, myocardial infarction, fractured ribs, and arthritis involving the bilateral ankles, knees, feet, and hands. It needs to be noted that he was on Plavix (clopidogrel) from a previous right coronary artery stent placement. Past surgical history included right ankle fusion, cervical spine surgery, coronary angioplasty with stent placement, tooth extractions, and childhood tonsillectomy and adenoidectomy. Other current medications included acetaminophen, atorvastatin, docusate, famotidine, gabapentin, ibuprofen, iopamidol, lisinopril, metoprolol succinate extended release, sublingual nitroglycerin, oxycodone, senna, and trazodone. He had no known drug allergies. He reported smoking cigars and drinking alcohol socially and denied recreational drug use.

Chest radiography showed no acute cardiopulmonary or bony abnormality. Contrast-enhanced CT of the chest, abdomen, and pelvis showed a large subcutaneous hematoma overlying the left pectoralis muscle with a small focus of active extravasation, a nondisplaced anterior left third rib fracture, and retrosternal blood products without evidence of a displaced sternal fracture (Figures 1, 2). No definite acute visceral posttraumatic findings were identified in the abdomen or pelvis.

**Figure 1:**
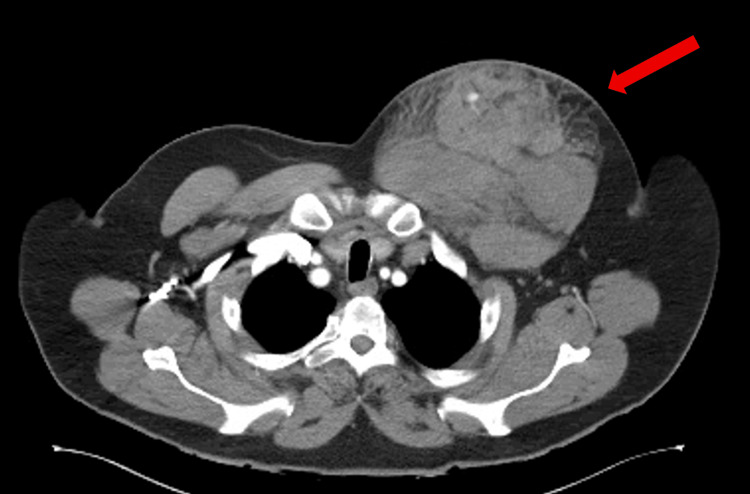
Contrast-enhanced CT chest (axial view) demonstrating a large subcutaneous hematoma overlying the left pectoralis muscle (red arrow) with a small focus of active extravasation.

**Figure 2:**
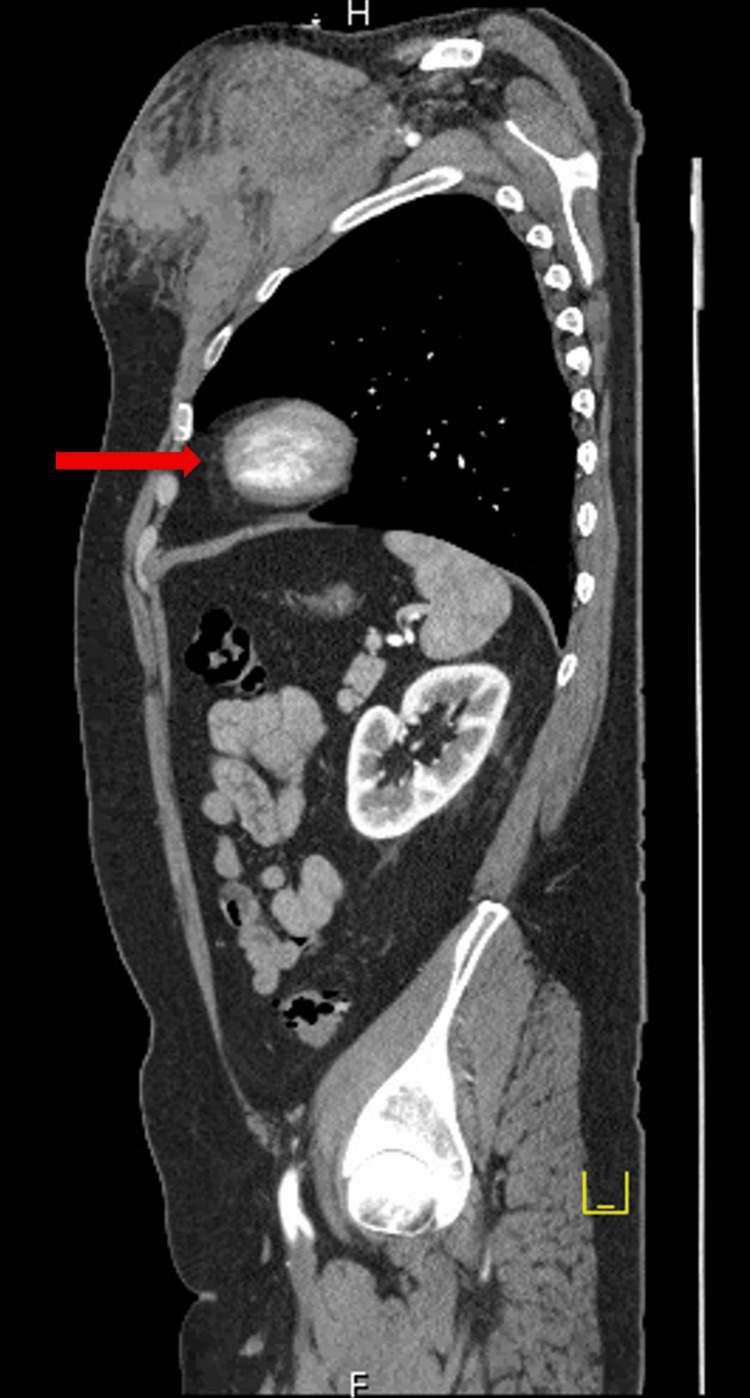
Contrast-enhanced CT (sagittal view) demonstrating a large subcutaneous hematoma overlying the left pectoralis muscle (red arrow) with a small focus of active extravasation.

CT of the head showed no acute intracranial abnormality. CT of the cervical, thoracic, and lumbar spine with reconstruction showed no acute spine fracture or traumatic malalignment. Radiographs of the left knee, tibia, fibula, and wrist were negative for acute fracture. Left subclavian angiography demonstrated unremarkable left subclavian and axillary arteries. The left vertebral artery and origin of the left IMA were unremarkable. Selective angiography of the left IMA identified a small bleeding branch corresponding to the focus of extravasation seen on CT.

During the ED stay, hemoglobin and hematocrit decreased to 10.5 g/dL and 29.8%, respectively. Systolic blood pressure decreased to the 80s (mmHg). Clopidogrel was held, and intravenous fluids were administered. The patient did not require a blood product transfusion. Vital signs were monitored over the next two hospital days, during which his blood pressure normalized.

Coil embolization was performed to control active hemorrhage originating from a branch of the left IMA. Under ultrasound guidance, right common femoral artery access was obtained, and a 5-French sheath was placed. A catheter was advanced into the left subclavian artery, where angiography confirmed active extravasation from a branch of the left IMA (Figure 3). Selective catheterization of the IMA was achieved using a microcatheter system. After initial difficulty accessing the bleeding branch, successful distal cannulation was obtained, and the vessel was embolized using multiple Hilal and Vortex microcoils. Post-completion angiography demonstrated resolution of active bleeding (Figure 4). Hemostasis at the access site was achieved with a MYNX™ closure device (Cordis, Miami, Florida, United States).

**Figure 3:**
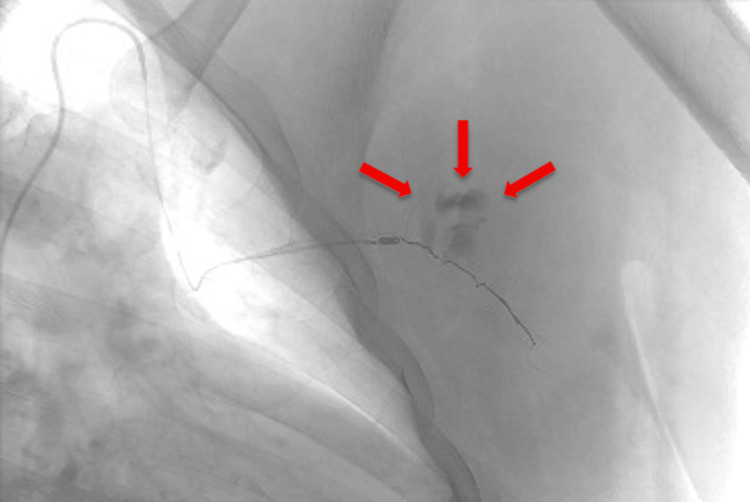
Pre-embolization angiogram of the left internal mammary artery (IMA) demonstrating an active bleeding branch (red arrows).

**Figure 4:**
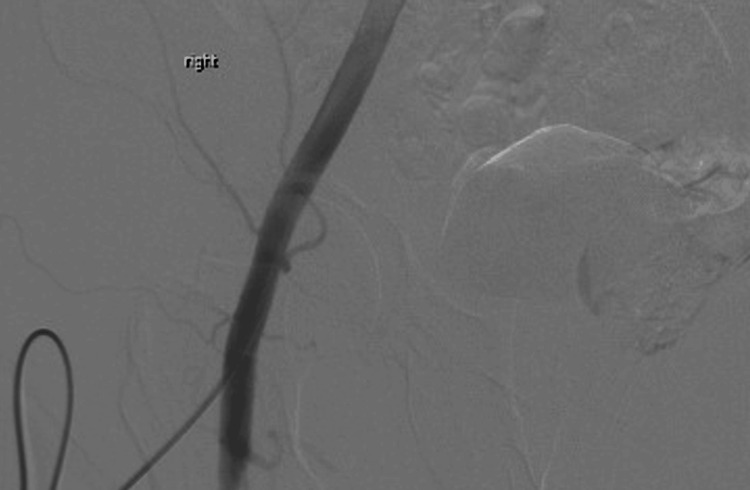
Post-embolization angiogram showing occlusion of left internal mammary artery (IMA) and resolution of active extravasation.

The patient was admitted overnight for close monitoring of blood pressure and serial hemoglobin and hematocrit values. Cardiology was consulted regarding his coronary stent and temporary interruption of clopidogrel. Echocardiography was ordered to further evaluate the retrosternal hematoma. Transthoracic echocardiography showed normal left ventricular systolic function, with an ejection fraction of 60-64%. Contrast was used to enhance endocardial border detection. Left ventricular diastolic dysfunction was present, consistent with pseudonormalization (grade II). The right ventricle had normal size, wall thickness, and systolic function.

The patient remained neurologically and hemodynamically stable. Hemoglobin levels stabilized, and clopidogrel was restarted on hospital day 2. He was evaluated by physical and occupational therapy and cleared for discharge home. At discharge, residual ecchymosis of the skin overlying the injury was visible (Figure 5). One week later, the patient followed up in the trauma clinic, primary care clinic, and cardiology clinic. His left chest wall swelling had significantly improved, and no post-procedural complications were noted.

**Figure 5:**
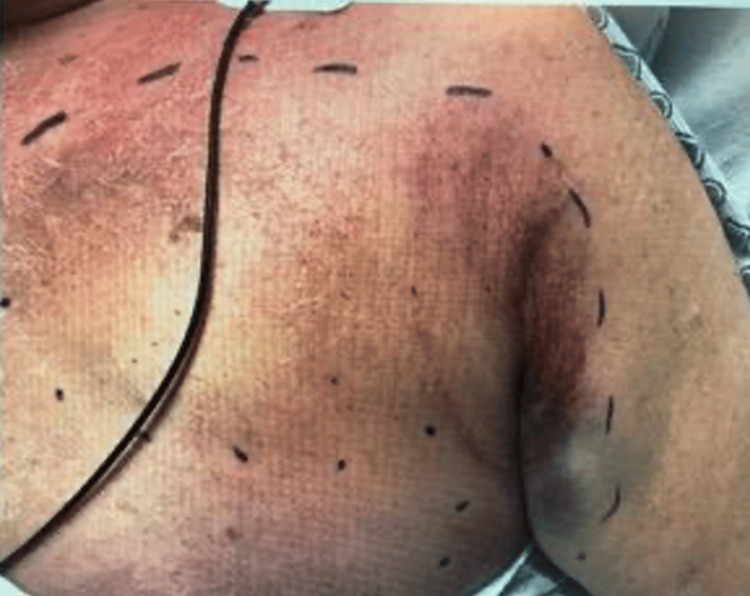
Residual ecchymosis from blunt trauma observed immediately before discharge.

## Discussion

The IMA branches from the subclavian artery, runs adjacent to the sternum posterior to the costal cartilage, and terminates in the sixth or seventh intercostal space, where it becomes the superior epigastric and musculophrenic arteries. This location, along with the rich collateral network of thoracic vessels, contributes to the risk of vascular injury after blunt anterior chest trauma. IMA injuries are rare because the artery is relatively well protected, and only a limited number of cases have been reported. These injuries most commonly result from automobile or motorcycle accidents but may also occur after relatively minor trauma [4]. IMA injuries are more common in men and more frequently involve the left IMA, as in this patient. Hemorrhage from IMA injury most commonly manifests as anterior mediastinal hematoma, hemothorax, pseudoaneurysm, arteriovenous fistula, or extrapleural hematoma [4-6].

The arterial blood supply of the breast is provided by several sources. The IMA arises from the first portion of the subclavian artery and provides approximately 60% of the total blood supply through its perforators [7]. These perforators course through the chest wall and supply the medial aspect of the breast [8]. The lateral thoracic artery, thoracoacromial artery, and vessels of the serratus anterior supply the remaining breast vasculature [9].

IMA injury from blunt chest trauma is typically associated with other injuries, including sternal or rib fractures, other vascular injuries, lung contusions, and neurologic changes related to hypoperfusion [10]. These associated injuries may distract attention from the IMA injury and delay diagnosis and treatment.

IMA injuries can cause life-threatening hemorrhage because the vessel is deep and not amenable to external compression, allowing bleeding to continue unchecked. Blood can rapidly accumulate under arterial pressure within the thoracic cavity, particularly the mediastinum or pleural space, which can accommodate several liters before clinical signs become apparent [11]. In addition to hemorrhagic shock and hypoperfusion, compressive complications such as cardiac tamponade may occur [1,6]. Depending on the location of bleeding and the extent of associated vascular injury, hemorrhage may range from self-limited to fatal. In a literature review of 49 cases with IMA injuries over 37 years, Chen et al. retrospectively reviewed 29 cases with complete reports and reported that nearly half of the patients presented with shock [4]. Other patients have presented with nonspecific symptoms, such as dizziness, anterior chest pain, or chest tightness [5]. Because presentations and trauma severity vary, diagnosis is often missed on initial evaluation and may be discovered later during operative repair of other injuries. In cases such as this one, in which relatively minor trauma resulted in IMA injury, timely diagnosis is essential. CT is commonly used in trauma evaluation and can effectively identify vascular injury and active bleeding [6]. However, CT may not always identify the culprit vessel. In these cases, angiography should be performed to confirm and localize the bleeding source. Active contrast extravasation on CT or angiography is a direct indication for endovascular or surgical intervention [12].

Treatment selection for IMA injury is multifactorial and depends on hemodynamic status, associated injuries, institutional resources, and the feasibility of endovascular access. In their literature review of IMA injury after blunt chest trauma, Chen et al. found that embolization was successful in 91.6% of patients, compared with 66% success for surgical ligation; however, because chest trauma is often associated with additional injuries, 45% of patients required surgical intervention to control bleeding [4]. Another study of 12 patients with acute IMA injury treated with transcatheter embolization demonstrated 100% technical success, defined by complete occlusion of each lesion [13]. Expert clinical judgment remains essential because many patients with IMA injury have associated injuries that may require operative management. In this patient, isolated vascular injury, transient hemodynamic response, and clopidogrel use supported endovascular embolization as an appropriate emergent treatment strategy. Preservation of future surgical options is also clinically relevant because the IMA is commonly used for coronary artery bypass grafting owing to its low anatomic variability and long-term patency [14].

## Conclusions

IMA injury following blunt chest trauma is uncommon but can lead to rapid hemodynamic deterioration when diagnosis is delayed. As demonstrated in this case, patients may initially present with mild symptoms, stable vital signs, and nonspecific findings despite significant vascular injury. Clinicians should maintain a high index of suspicion for IMA injury in patients with blunt anterior chest trauma, enlarging chest wall hematoma, hypotension, or active extravasation on imaging. Early use of contrast-enhanced CT or CT angiography is important because these modalities can detect active bleeding, guide angiographic localization, and inform management.

In this case, endovascular embolization provided effective hemorrhage control and avoided the morbidity associated with open surgical repair. Embolization appears to offer advantages in appropriately selected patients, including rapid hemostasis and reduced physiologic burden. Surgical intervention remains necessary in patients with refractory instability or associated intrathoracic injury; however, this case supports angiographic embolization as a first-line treatment option in stable or transiently responsive patients with isolated IMA injury.
